# Challenges Faced by Talented Students in Medical Sciences Education: A Phenomenological Study

**DOI:** 10.34172/aim.34288

**Published:** 2025-07-01

**Authors:** Houra Ashrafifard, Hossein Karimi Moonaghi, Raheleh Gharibnavaz, Eshagh Ildarabadi

**Affiliations:** ^1^Department of Medical Education, School of Medicine, Tehran University of Medical Sciences, Tehran, Iran; ^2^Nursing and Midwifery Care Research Center and Medical Sciences Education Research Center, Mashhad University of Medical Sciences, Mashhad, Iran; ^3^Deputy of Education, Mashhad University of Medical Sciences, Mashhad, Iran; ^4^Department of Nursing, Esfarayen Faculty of Medical Sciences, Esfarayen, Iran

**Keywords:** Education, Medical, Qualitative research, Students, Universities

## Abstract

**Background:**

Talented students are among the key human resources in society, and educational and university authorities bear the responsibility of addressing the potential challenges they face. Therefore, identifying these challenges is essential.

**Objective:**

This research aimed to identify the challenges experienced by talented students and to propose possible solutions to help managers and planners design effective interventions.

**Methods:**

The participants were 50 talented students from healthcare sciences. A phenomenological approach was employed using Colaizzi’s seven steps method for data collection and analysis. Structured interviews were conducted with the students selected through purposive sampling.

**Results:**

Data analysis revealed the central theme of the "narrow alley of talents" accompanied by six main themes: "motivational slope", "striving for improvement", "uneven university path", "social lens", "family umbrella", and "the bright side of talents", each comprising several subthemes.

**Conclusion:**

The challenges faced by talented students can be mitigated and their competencies enhanced through tailored educational opportunities and services, counseling, and engagement of external motivational and support resources.

## Introduction

 The concept of “talent” encompasses general mental abilities, special academic aptitudes, creative thinking, exceptional skills in the arts, and leadership skills. Although there is no universally accepted definition, the US federal government has identified these core elements to guide planning for talented individuals, emphasizing their cognitive, emotional, and social potential.^1^ Talented or gifted students are a valuable asset to the society, and it is the responsibility of educational institutions and universities worldwide to ensure their appropriate education and development.^2^ However, increasing demand and retention challenges pose significant issues for talent management in higher education, highlighting the need for more effective strategies in managing academic staff. Efficient talent management can enhance the productivity of talented students.^3^ Failure to recognize and address their needs may lead to adverse outcomes. It is essential to consider the unique challenges this group faces,^4,5^ as not all talented students are able to fully realize their potential.^6^ Moreover, elite migration results in loss of specialized human capital, exacerbating disparities between host and source countries. McKinsey refers to this as a “war and competition to acquire talent”.^7^ Consequently, the investigation of the challenges faced by talented students is a critical priority. Changizi et alcompared the perspectives of talented and other students regarding educational issues at Arak University of Medical Sciences. The study found that talented students had different perspectives on challenges from others, such as neglect of individual talents, lack of enrichment programs based on differences, insufficient academic counseling, and inadequate awareness of educational regulations among students and advisors.^8^ Similarly, Awandu identified several challenges talented learners in Laikipia Central District face such as lack of motivation, inappropriate teaching methods, an unsuitable curriculum, exploitation, mistreatment, and social isolation.^9^

 Nations increasingly recognize the importance of nurturing talented students to drive innovation, development, and societal progress. These individuals are considered national assets, contributing to civilization and prosperity. Consequently, developed countries often invest heavily in supporting talented students by providing necessary resources and opportunities to help them reach their potential.^10^ Research shows a link between education and economic growth, particularly in developing countries. Many of these nations are now focusing on talent development through improved tertiary education and research, aiming to build the intellectual capital needed for national advancement.^11^ As a developing country, Iran acknowledges the transformative role of universities in achieving this goal.

 According to regulations from Iran’s Ministry of Health, students eligible for the Talented Committee must meet one or more of the following criteria: national exam rank within the top 500; recipients of gold, silver, or bronze medals in Olympiads and science festivals; holders of registered inventions or patents; top 10 ranks in graduate school entrance exams; top 1% of students in each field with a grade point average (GPA) above 17 (for associate and bachelor’s degrees); or above 16 (for master’s degrees and above), and no disciplinary convictions.^12^ Iran’s academic community has long debated the importance of supporting talented students. Failing to do so can result in significant talent loss due to migration or underutilization.^4^ Brain drain remains a major challenge in developing countries like Iran, which ranks high globally in talent migration. Many medal-winning students emigrate to countries such as the United States, Canada, and those in Western Europe, causing substantial annual costs for the Iranian government.^7^

 To foster positive change, it is crucial to gather foundational knowledge about the challenges and lived experiences of talented students. This information can help reinforce supportive factors, mitigate obstacles, and harmonize efforts to address their needs. Understanding the students’ perspectives is essential for designing appropriate support mechanisms that enable them to succeed.^13^ Given the limited research on the challenges faced by talented students in healthcare education, this study aims to explore their perceptions and experiences in greater depth.

## Materials and Methods

###  Type of the Study

 This study employed a phenomenological approach to explore the students’ experiences and the challenges they encountered. Phenomenological research examines the individuals’ experiences through methods such as interviews, narratives, or observations, with an emphasis on understanding conscious elements including judgments, perceptions, and emotions.^14^ This approach aims to uncover the meanings embedded in these experiences and the insights they provide into human consciousness and lived reality.^15^

###  Participants

 A purposive sampling approach was employed to select 50 participants for this study. A list of eligible students including their names and contact information was obtained from the university’s talent unit. To ensure a diverse sample, our selection criteria encompassed various factors such as field of study, academic semester, living arrangements, marital status, gender, and age. The demographic characteristics of the participants are presented in Table 1.

**Table 1:** Demographic Characteristics of the Participants

	**Male**	**Female**	**Mean Age**	**GPA**
School of Medicine	12	6	21.11 ± 0.87	16.97 ± 1.22
School of Health	3	3	22.17 ± 1.95	17.34 ± 1.02
School of Dentistry	3	4	21.2 ± 2.04	17.18 ± 1.02
School of Nursing & Midwifery	4	11	23.27 ± 3.13	18.30 ± 0.44
School of Para-medicine	2	2	21.75 ± 2.16	16.62 ± 0.49
Total	24	26	21.1 ± 2.35	17.51 ± 1.09

GPA, Grade point average.

###  Data Collection

 Semi-structured individual interviews were used to collect data through open-ended questions. The interview questions were as follows:

1. How did you feel when you were selected as a talented student?
2. Tell your story about the challenges you encountered in this field.
3. What challenges have you experienced in this field?
4. Explain your feelings and perceptions regarding joining the talent team.
5. What do you compare being a talent to?
6. What items are important to you? What are your requirements and challenges?
7. What are your experiences and perceptions about family, academic, and social factors associated with you?
8. If there are any additional problems, please state them. Could you provide instances to help me understand your point better? If you want to discuss anything else, feel free to let me know.

Before the interviews began, participants were provided with a thorough explanation of the study’s objectives and potential applications. Verbal informed consent was then obtained from all participants. They were made aware of their right to withdraw from the study at any time. To ensure confidentiality, participants’ names were replaced with letters in all research reports. No incentives were offered for participation. Interviews were conducted remotely via WhatsApp and Telegram, with each session lasting approximately 40 to 60 minutes. Participants followed a structured interview protocol, responding to questions sequentially through voice messages. Probing questions were also used as needed throughout the interview process. Data collection continued until theoretical saturation was reached.^16^

###  Data Analysis

 Colaizzi’s seven-step method was applied as follows:

1. The researchers familiarized themselves with the collected data by reading the descriptions and findings of the participants.
2. Significant sentences, phrases, and statements related to the phenomenon were identified and extracted.
3. Meanings were formulated from the extracted statements, with researchers taking care to minimize the influence of personal preconceptions.
4. The formulated meanings were categorized into themes. Researchers referred back to the original statements and removed any inconsistent data.
5. The researcher synthesized the themes into a comprehensive description of the phenomenon.
6. This comprehensive description was then condensed into a single sentence, including only the significant structural elements of the phenomena.
7. To seek verification, the participants were asked to review the results of the analysis.^17^ The findings were shared with them for feedback on whether the interpretations reflected their experiences. Feedback was obtained from 38 participants, with clarifications or completions made in two cases.

###  Data Validity

 To ensure reflexivity and bracketing (Epoche), researchers made a conscious effort to remain aware of their biases, preconceptions, and personal perspectives. They systematically documented and reflected on the biases and assumptions they identified. Throughout the research process, they adapted their understanding based on their interactions with the data and shared their perspectives with each other to uncover any hidden biases. Data analysis and interpretation of findings were conducted with an awareness of personal biases.

## Results

**Figure 1:**
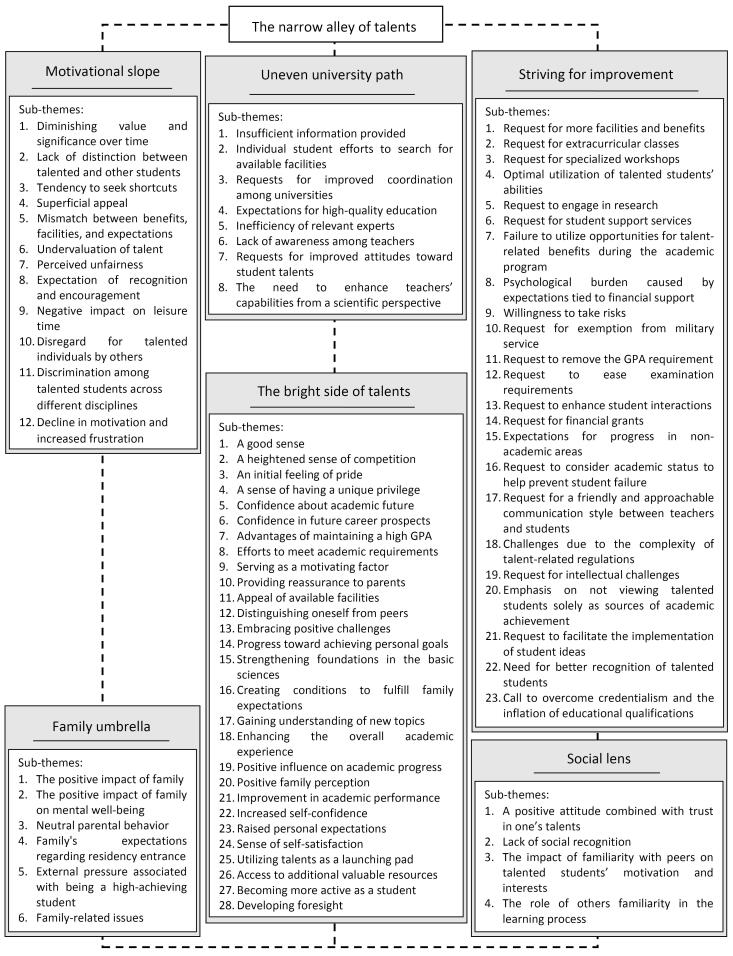
Main Theme, Themes, and Sub-themes

 A total of 1175 initial codes, 185 secondary codes, 81 sub-themes, and six themes were identified. Data analysis revealed the “narrow alley of talents” as the central theme, accompanied by six main themes: “motivational slope”, “striving for improvement”, “uneven university path”, “social lens”, “family umbrella”, and “the bright side of talents”. Each theme encompassed several sub-themes. Figure 1 provides an overview of the central theme, main themes, and their associated sub-themes.

###  Motivational Slope

 Talented students often feel that their significance diminishes over time, perceiving little to no distinction between themselves and their peers. This perception has led to a decline in motivation and a rise in frustration among many of them. As one student explained, *“Initially, I felt good about it, but once I entered the university, that feeling began to fade. The excitement and enthusiasm I once had gradually diminished, and now they continue to decline.” *Another student shared,*” It was unexpected at first, but over the following semesters, it became normal. I realized that nothing special was happening. There’s no particular sense of superiority between me and the other students.”* While the label of “talented” may carry superficial appeal and occasionally offer shortcuts to advancement, many talented students have faced increasing challenges in accessing the resources and opportunities intended for them. This has contributed to their growing sense of frustration. Nevertheless, some interviewees noted that teachers’ recognition and attention in the classroom can serve as a motivating factor.

 One student stated, *“Talent is not worth fighting for.”* Another remarked *“Being part of a talent program should be a secondary goal, not the main one*.” Yet another stated, *“Some teachers motivate talented students simply by the importance they place on them.”*

###  Striving for Improvement

 Talented students expressed the need for greater support to improve their circumstances, including considerations such as exemptions from military service. They emphasized that importance of establishing specific criteria for scholarship eligibility. Additionally, they highlighted the necessity of creating opportunities for idea generation and fostering conducive environments for research activities. Students also called for improved access to research channels and innovative research topics. They suggested that clear regulations should be developed to support and enhance their overall academic and professional development. One student identified *“exemption from military service and direct admission into residency programs”* as key benefits of being recognized as talented. Another remarked,* “These issues are personally important to me, especially since I am currently studying outside my home city. I need to find ways to address these needs within academic communities... For instance, various workshops and conferences held at the university level can be very helpful to me.”*

###  Uneven University Path

 The students’ experiences highlight inefficiencies among university staff and a lack of awareness among teachers. One student stated, *“There is very little information about the talents. There is no one who explains it clearly.”* Additionally, there is insufficient understanding of the regulations and resources available for talented students, which hinders efforts to address their needs effectively. Enhancing teachers’ skills in engaging with talented students is essential. The education system for high-achieving students should be more efficient and responsive to their expectations. Reflecting this, one student emphasized the need to *“hold briefing meetings for the teachers”,* while another identified *“the failure of the university to announce the regulations compiled in this regard” *as a significant challenge.

###  Social Lens

 Talented students often receive positive feedback from those around them, including their teachers both within and outside the university — feedback they regard as valuable. These students are typically recognized for having reached this level of achievement through consistent and frequent studying. One student remarked, *“My classmates often label me a nerd. They believe that’s the only reason for my talent.”* These students tend to gain greater trust from others, meet teachers’ expectations more readily, and consequently receive increased attention. However, this can sometimes provoke negative reactions from peers. As a result, some students may feel pressured to hide their talents. One student mentioned experiencing *“changes in the family attitudes toward the student” and “dissatisfaction among other classmates”,* while another noted that *“Inappropriate treatments undermines the dignity of students”. *

###  Family Umbrella

 Family support and encouragement provided students with a sense of peace, acting as a protective shield. This group of students found the family’s role to be both empowering and beneficial to their academic pursuits. Supportive and engaged families are essential to students’ educational success, whereas indifferent families often fail to contribute meaningfully to their children’s learning. Encouragement and feedback from family members not only foster academic motivation but also support students’ psychological well-being. Some families expressed pride in their children’s talents, encouraging them to pursue residency. One student highlighted *“the effective role of motivation and encouragement provided by the family during times of hopelessness and exhaustion”* and *“the positive words offered by family members”. *

###  The Bright Side of Talents

 For this group, personal satisfaction and fulfilling both their own and their families’ expectations as talented students are of great importance. They value having special privileges and view guaranteed future success in academics and careers as essential. These students believe that talent plays a critical role in achieving future success. One student remarked*, “It actually creates a positive kind of stress that serves as a reminder to always strive for more and to study hard in order to achieve high grades. This is a positive challenge.”* They set high expectations for themselves and feel a strong sense of pride, which significantly influences their future achievements. One student referred to the effects of *“perfectionism” *in this context, while others emphasized* “satisfaction with individual performance” *and *“greater perseverance and effort”. *

## Discussion

 This study aimed to illustrate the challenges faced by talented students in healthcare education. The findings highlight three key concepts: motivation, social dynamics, and talent development.

 Talented students in this study reported a decline in motivation over time, leading them to believe that there is no meaningful distinction between themselves and other students. Research suggests that lack of motivation is a complex issue that can prevent some students from reaching their full potential.^18^ Zbainos and Kyritsi emphasized the need for a flexible and motivating educational system tailored specifically to the needs of talented students.^19^ Consistent with the present findings, previous research has identified several motivational barriers faced by talented students. These include structural challenges, such as vague regulations, lack of program differentiation, unclear organizational goals, and poor integration of research and academic components. Additionally, operational issues (including weak communication within the talent office, limited student-staff interaction, unfulfilled promises by officials, and ineffective recruitment strategies) further hinder motivation.^20^ The study highlights that motivational barriers often evolve and tend to intensify after students enter the university. A decline in motivation may result from the perception that their talents are not recognized or adequately nurtured, thereby exacerbating the disconnect between their high school and university experiences. While students acknowledged their abilities, they also stressed the importance of external validation and rewards. According to self-determination theory (SDT), talented students face challenges when their basic psychological needs (autonomy, competence, and relatedness) are not met in the educational system. SDT highlights that these needs are essential for students’ motivation and optimal functioning. When unmet, students may rely on external or controlled motivations. Support from parents, teachers, and peers is crucial in fulfilling these needs, which are interconnected and should be addressed together.^21^

 The social dynamics experienced by talented students in this study can be better understood through several key aspects grounded in Social Identity Theory (SIT). SIT, a concept in social psychology, helps explain how individuals choose and internalize social group memberships. It outlines three key principles: (1) An individual’s self-concept is partly derived from their group affiliations. (2) The focus is on the collective self, shaped by group membership and interactions with others in the group (linked to social categorization theory). (3) Social identification influences behaviors within and between groups (related to intergroup behavior). Social categorization theory supports SIT by describing how people mentally organize social groups and form identities based on shared norms, values, and emotional significance.^22^ Students’ experiences revealed inefficiencies among university staff, lack of awareness among teachers, and ignorance of regulations and resources for talented students. These challenges can negatively impact the social identity of such students. In fact, the findings of this study suggest that issues commonly perceived as purely academic can also influence the identity formation of talented students. Since these students often view themselves as distinct from their peers, it is important to pay special attention to the sources of information they rely on. Based on the presented theory, we believe that such students may construct part of their identity within the university setting through exposure to instructions, regulations, and guidance from those around them, including university staff. On the other hand, Social identity theories explain that people evaluate themselves and others based on group norms. Those who align with these norms are perceived as in-group members, while those who differ are considered as out-group members. People often highlight these group differences to boost their own group’s status.^22^ Two points from the study illustrate this theory: First, a notable finding is the sometimes negative perception of peers toward talented students, which may pressure them to conceal their abilities. This dynamic can hinder their social identity formation. Second, many students report struggling with perfectionism, which appears to be linked to their efforts to construct a strong and positive social identity. It is important to note that these students are eager to engage in various activities to demonstrate their abilities and enhance their social image. While they prefer to complete tasks independently,^23^ it remains essential for universities to provide them with appropriate emotional and social support.

 Talented students emphasized various aspects of their interactions with others. They reported receiving positive feedback from both teachers and peers, both within and outside the university. However, they also noted experiencing negative reactions from some classmates. Additionally, they highlighted the significant role that families play in shaping their experiences. These factors indicate that talented students cannot be fully understood solely from the university’s perspective. Bronfenbrenner’s Ecological Systems Framework emphasizes the importance of studying individual development through interactions across multiple environments. In contrast, research on underrepresentation in talented programs often focuses too narrowly on single settings, missing the broader, interconnected factors that are involved.^24^ Our findings suggest that universities should provide appropriate and consistent socio-emotional support for talented students, considering their high emotional intelligence, community engagement, understanding of social values, and moral sensitivity.^25-27^ Consistent with our findings, many educators lack the necessary experience to meet the complex needs of talented students,^26^ highlighting the importance of mandatory and ongoing training in talented education.^28^ Reis et al identify key factors that support talented students’ success, including encouraging adults, positive peer relationships, extracurricular opportunities, and a stable home environment.^29^ Four main educational needs are emphasized: a challenging curriculum, access to intellectual peers, supportive parenting, and understanding adults.^26^ Moreover, families play a crucial role in how talented students develop their abilities and cope with both emotional and academic stress.^30-32^ Recognizing this role is essential to effectively support talented students in overcoming challenges.

 Finally, in terms of talent development, personal satisfaction and the fulfillment of both individual and family expectations were important for talented students. Programs designed for these students should have clearly defined goals that align with their specific needs. The present study suggests that the needs for a clear definition of talent in education, identifying it as cognitive achievement across both academic and non-academic areas. Talent varies across individuals, with each student showing strengths in specific domains.^33^ Therefore, flexible, personalized support is essential to help talented students thrive.^34,35^ Continuous practice, problem-solving, and varied instructional strategies are key to developing their abilities.^26,32,36^ Supporting their intellectual ideas, values, and goals strengthens their cognitive development and fosters lifelong learning.^37^ However, educational programs often fall short of meeting these students’ needs.^34,38^ Reis suggests that effective programs should be high-quality, comprehensive, practical, consistent, and address both academic and emotional aspects.^39^

 A notable feature of our study was the use of social media to collect qualitative data. Over the past decade, advancements in digital technologies have significantly influenced qualitative research. These developments have transformed how researchers access and share information, communicate with participants, and collect and disseminate data. For instance, qualitative data can now be gathered through instant messaging, email, or online/video chats across various platforms.^40^ Research has shown that interviews conducted via telephone, face-to-face, video, and email may yield different numbers of codes; however, the substance and content remain consistent across these modes.^41^ In our study, interviews were conducted through voice messages on social media. When comparing voice and video interviews, studies indicate that video interviews foster a stronger sense of connection between interviewer and interviewee than telephone interviews. This increased rapport often facilitates more open and detailed expression of thoughts on the research topic.^41^ The literature presents mixed findings on online versus in-person interviews. While audio-only formats can limit the ability to observe non-verbal cues, potentially creating a sense of disconnection and reducing depth,^42^ other studies report no significant differences between face-to-face and online interviews. In fact, some participants feel more comfortable sharing in online settings due to the physical distance, as they are not required to host researchers in their personal or professional spaces.^43^

## Implications

###  Short-term Institutional Interventions

- The findings of this research highlight the varied experiences of talented students. By understanding the challenges these students face, educators can enhance their potential and maximize the return on educational investment.
- By addressing the challenges faced by talented students, teaching methods can be improved to enhance their abilities.
- Learning environments can be improved to better support talented students by addressing challenges and removing barriers that affect their unique needs and abilities.

###  Long-term Policy Recommendation

- Understanding the challenges faced by talented students can aid administrators in educational policy-making.

## Limitations

 One limitation of this study is its focus on undergraduate students, which restricts the generalizability of the findings to other academic levels. Furthermore, the study used purposive sampling, which limits generalizability.

## Further Directions

 This study focused on the experiences of healthcare students in Iran. Future research could include comparative cross-national studies to explore the concept of challenges from the perspective of students from various cultural backgrounds. Additionally, longitudinal studies could be designed to assess the effectiveness of strategies aimed at overcoming challenges and barriers, to improve the education of talented healthcare students.

## Conclusion

 Inadequate university support, emotional and social challenges in campus interactions, and limited access to the benefits of talent-based programs contribute to what can be described as the “narrow alley of talents.” These challenges can be mitigated, and the efficiency and potential of talented students enhanced, through provision of tailored educational opportunities, dedicated counseling services, and access to external motivational and support resources. The findings of this study can inform the efforts of students, educators, and educational managers in planning and implementing strategies to empower talented students, who represent a vital asset to the educational system.
